# Subjective mental health, incidence of depressive symptoms in later life, and the role of epigenetics: results from two longitudinal cohort studies

**DOI:** 10.1038/s41398-020-00997-x

**Published:** 2020-09-21

**Authors:** Laura Perna, Yan Zhang, Pamela R. Matias-Garcia, Karl-Heinz Ladwig, Tobias Wiechmann, Beate Wild, Melanie Waldenberger, Ben Schöttker, Ute Mons, Andreas Ihle, Matthias Kliegel, Lars Schwettmann, Annette Peters, Hermann Brenner

**Affiliations:** 1 German Cancer Research Center (DKFZ) – Division of Clinical Epidemiology and Aging Research, Im Neuenheimer Feld 581, 69120 Heidelberg, Germany; 2 Department of Translational Research in Psychiatry, Max Planck Institute of Psychiatry, 80804 Munich, Germany; 3 Technical University of Munich, TUM School of Medicine, Ismaninger Straße 22, 81675 München, Germany; 4 Helmholtz Zentrum München – Research Unit Molecular Epidemiology – Ingolstädter, Landstraße 1, 85764 Neuherberg, Germany; 5 Helmholtz Zentrum München – Institute of Epidemiology, Ingolstädter Landstraße 1, 85764 Neuherberg, Germany; 6 Department of Psychosomatic Medicine and Psychotherapy, Klinikum rechts der Isar, Technische Universität München, Langerstraße 3, 81675 München, Germany; 7 University of Heidelberg – Department of General Internal Medicine and Psychosomatics, Medical University Hospital, Im Neuenheimer Feld 672, 69120 Heidelberg, Germany; 8 University of Heidelberg – Network Aging Research (NAR), Bergheimer Straße 20, 69115 Heidelberg, Germany; 9 German Cancer Research Center (DKFZ) – Cancer Prevention Unit, Im Neuenheimer Feld 280, 69120 Heidelberg, Germany; 10 University of Geneva – Department of Psychology, Boulevard du pont d’ Arve 40, 1211 Geneva, Switzerland; 11 University of Geneva – Center for the Interdisciplinary Study of Gerontology and Vulnerability, Boulevard du pont d’ Arve 40, 1205 Geneva, Switzerland; 12 University of Lausanne – Swiss National Centre of Competence in Research LIVES – Overcoming vulnerability: Life course perspectives, Géopolis Building, 1015 Lausanne, Switzerland; 13 Helmholtz Zentrum München – German Research Center for Enviromental Health (GmbH) – Institute of Health Economics and Health Care Management, Ingolstädter Landstraße 1, 85764 Neuherberg, Germany; 14 Department of Economics, Martin Luther University, Halle Wittenberg, 06099 Halle, (Saale) Germany; 15 Institute for Medical Information Sciences, Biometry and Epidemiology, Ludwig-Maximilians Universität, Marchioninistr. 15, 81377 Munich, Germany

**Keywords:** Predictive markers, Molecular neuroscience

## Abstract

The role of self-perceived general health in predicting morbidity and mortality among older people is established. The predictive value of self-perceived mental health and of its possible biological underpinnings for future depressive symptoms is unexplored. This study aimed to assess the role of mental health-related quality of life (HRQOL) and of its epigenetic markers in predicting depressive symptoms among older people without lifetime history of depression. Data were based on a subgroup (*n* = 1 492) of participants of the longitudinal ESTHER study. An epigenome-wide association study (EWAS) of mental HRQOL was conducted using DNA from baseline whole blood samples and logistic regression analyses were performed to assess the predictive value of methylation beta values of EWAS identified CpGs for incidence of depressive symptoms in later life. The methylation analyses were replicated in the independent KORA cohort (*n* = 890) and a meta-analysis of the two studies was conducted. Results of the meta-analysis showed that participants with beta values of cg27115863 within quartile 1 (Q_1_) had nearly a two-fold increased risk of developing depressive symptoms compared to participants with beta values within Q_4_ (OR_Q1vsQ4 _= 1.80; CI 1.25–2.61). In the ESTHER study the predictive value of subjective mental health for future depressive symptoms was also assessed and for 10-unit increase in mental HRQOL scores the odds for incident depressive symptoms were reduced by 54% (OR 0.46; CI 0.40–0.54). These findings suggest that subjective mental health and hypomethylation at cg27115863 are predictive of depressive symptoms, possibly through the activation of inflammatory signaling pathway.

## Introduction

Depression is a common mental disorder and a leading cause of disability worldwide^1^. Prevalence of depression decreases in older age, but especially among people older than 65 years (late-onset depression) depression is more closely associated with suicide and decreased physical, cognitive, and social functioning than early-onset depression^2^. Subthreshold depressive disorders, whose impact on health status is comparable to full-blown depressive episodes^3^, also have a high prevalence among older adults^2,4^.

The exact molecular mechanisms underlying depression are still unclear, but a growing body of evidence suggests that DNA methylation is a candidate mechanism contributing to explaining the molecular basis of late-onset depression^5^. Yet, there is a lack of longitudinal studies investigating the predictive value of DNA methylation in depression with most studies being cross-sectional and having a small sample size. Also, epigenetic markers of depression have been mainly investigated among people with manifest depression. However, in order to better understand mechanisms leading to mood disorder and to be able to predict risk of depression among healthy people one would need to capture markers relevant to depression before the pathology becomes apparent.

The role of self-perceived general health in predicting morbidity and mortality among older people is established^6,7^ and there is an increasing number of studies showing the relevance of subjective cognitive decline for risk of dementia^8^. Less work has been conducted on the role of subjective mental health and risk of mood disorder among healthy older individuals. Here, we test the hypothesis that self-perceived mental health and its possible epigenetic underpinnings allow predicting depressive symptoms among older people. The Mental Component Summary of the Short-Form 12 Health Survey (MCS-12) measures self-perceived mental health-related quality of life (mental HRQOL) by assessing important domains of mental well-being, such as psychological distress, vitality, and emotional problems^9^. Hence, the MCS-12 could be used as an instrument to explore whether self-perceived mental health and its epigenetic signature can predict depressive symptoms over the years among older people.

This study aimed to assess whether mental HRQOL is predictive of depressive symptoms and to perform an epigenome-wide association study (EWAS) of mental HRQOL to explore whether the resulting epigenetic markers would predict depressive symptoms among older people without lifetime history of a physician diagnosis of depression and free of depression at baseline over an observational period of 5 years.

## Method

The main analyses were conducted with the ESTHER longitudinal cohort initiated in 2000–2002 (baseline) in the German state of Saarland and including in total 9940 participants aged 50–75 years^10^. Part of the analyses were replicated in the independent KORA F4 study, which was initiated in 2006–2008 in the German region of Augsburg and included 3,080 participants aged 32–81 years^11–13^. The ESTHER study was approved by the Ethics Committees of the Medical Faculty of the University of Heidelberg and of the Physicians’ Board of Saarland. The KORA study was approved by the Ethics Committee of the Bavarian Medical Association. All participants of the ESTHER and KORA F4 study gave written informed consent.

Mental HRQOL was measured with MCS-12 scores of the Short-Form 12 Health Survey (4-week recall) administrated at baseline of the ESTHER study. MCS-12 scores were computed and missing items were handled using modified regression estimation methods as described by Maatouk and collegues^14^. MCS-12 scores can range from 0 to 100 with higher scores indicating better health. Depressive symptoms in the 5-year follow-up (2005–2007) of the ESTHER study were measured with the 15-item Geriatric Depression Scale^15^ (GDS-15). If ≤5 items of the GDS were missing (*n* = 108) the total score was prorated according to Yesavage^16^; if >5 items were missing the scale was invalidated (*n* = 95). Scores higher than 5 were considered as an indication for possible depressive symptoms warranting further depression screening^16,17^. In the KORA cohort the GDS-15 was not available but the Depression and Exhaustion Scale (DEEX)^18,19^ was measured in the follow-up of the KORA cohort (KORA FF4) in 2013–2014. The DEEX is a screening instrument used to identify exhausted and depressed mood among healthy individuals^19^. It combines eight items (irritability, inner tension, loss of energy, difficulty in concentrating, nervousness, fatigue, tiredness, anxiety) and total scores range from 0 to 24 with higher scores indicating worse mental health.

DNA methylation was quantified in both cohorts in baseline whole blood of a subgroup of participants (*N*_ESTHER_ = 2335, *N*_KORA_ = 1727) with Infinium HumanMethylation450K BeadChip (Illumina, Inc, San Diego, CA, USA). Details of methylation assessment have been reported previously for both cohorts^20,21^. In brief, 1.5 (KORA: 1.0) μg DNA (allocated in 96-well format with three random duplicate samples in each format as quality controls) was bisulfite converted, and 200 ng bisulfite-treated DNA was applied to the 450 K BeadChips following the manufacturer’s instruction. Raw data pre-processing and initial quality control was carried out following the CPACOR pipeline^22^. Samples with more than one control value below normal, or with sex mismatched with genetic sex were removed before data processing. Probes with detection *p*-value >0.01 (KORA ≥0.01), probes targeting the sex chromosomes, cross-reactive probes, and polymorphic CpGs^23^ were removed before quantile normalization, which was applied following stratification of the probe type into six categories according to probe type and color channel, using the R package limm^24^. Sample call rate threshold and CpG call rate threshold both were 95%. Houseman algorithms^25^ were used for estimating leukocyte distribution in both cohorts.

In the ESTHER cohort DNA methylation was measured separately in three independent subsets with no overlap among them one year apart from each other as already described previously in detail^10^ (Supplemental Fig. 1). EWAS analyses relating to mental HRQOL were performed in the ESTHER cohort separately in subset I (*n* = 1000) and subset II (*n* = 864) and an epigenome-wide meta-analysis of mental HRQOL based on subset I and subset II was conducted. First, a genome-wide significance threshold of FDR < 0.05 was applied, but none of CpGs remained significant after multiple testing correction. Hence, the more relaxed epigenome-wide significance cut-off point of *p*-value <1.0E-5 was used. The CpGs that reached the statistical significance were further validated in subset III (*n* = 471) and subsequently tested for a potential association with risk of depression both in the ESTHER and in the KORA study. Following the CPACOR pipeline, a principle component analysis (PCA) based on all 450 K methylation array control probes was performed, and the first 30 PCAs were included in the regression model as technical covariates. In the KORA cohort the first 20 PCAs were included. CpG annotation was carried out with the UCSC Genome Browser and the Roadmap Epigenome Browser^26^.

In the ESTHER cohort participants with information relating to a lifetime history of a physician diagnosis of depression (including baseline depression) were excluded (*n* = 246) and association analyses were performed with logistic regression models. In the KORA cohort information on lifetime history of depression was not available, but at baseline the Patient Health Questionnaire (PHQ-9) and the DEEX were administrated. Hence, all participants with baseline depressive symptoms that could indicate the presence of a depressive episode were excluded; specifically, KORA participants with a total score ≥10 of the PHQ-9 were excluded (*n* = 11). Furthermore, in regression models conducted with the KORA cohort the baseline DEEX score was additionally included as potential confounding variable.

The outcome variable was for both cohorts a binary variable indicating incident depressive symptoms (GDS score > 5^16,17^ in the ESTHER cohort and DEEX score ≥9 for men and ≥11 for women in the KORA study^27^). Participants with a GDS score ≤5 and with a DEEX sex-specific score <9 for men and <11 for women were considered to be free of depressive symptoms. The predictor variable was the statistically significant CpG site identified by the EWAS, and results are presented by increase in its methylation beta value by one standard deviation (SD). For easier interpretation, the methylation beta values were additionally divided into quartiles (Q) and odds ratios (OR) are also presented for quartiles 1 to 3 compared to the highest quartile.

Regression model 1 included for both cohorts the following covariates: age (continuous), sex, educational level (low: certificate of completion of compulsory basic secondary schooling or lower; middle: intermediate secondary school-leaving certificate, high: general qualification for university entrance or higher qualification), batch effects, and leukocyte distribution including the following cell types: B lymphocytes, CD8 T-cells (only ESTHER), CD4 T-cells, granulocytes, monocytes, natural killer cells. Model 2 was additionally adjusted for potential confounding variables available in both cohorts, including smoking (never, former, current), physical activity (ESTHER: ≥ 2 h of physical activity/week vs. inactive/<2 h of physical activity/week; KORA: ≥ 1 h of physical activity/week vs. <1 h of physical activity/week), body mass index (continuous), and lifetime history (KORA: baseline prevalence) of cardiovascular disease (myocardial infarction or stroke). The covariates included in the regression models were collected at baseline at the same time point as that used for the collection of blood DNA methylation. Furthermore, a random-effects meta-analysis combining the results of the ESTHER and KORA cohort was conducted. In the ESTHER study a logistic regression model was also calculated to investigate the predictive value of MCS-12 (per 10-units) for incident depressive symptoms among people with no lifetime history of depression.

Sensitivity analyses were conducted after additional exclusion of participants using antidepressants at baseline (Anatomical Therapeutic Chemical Code: N06A, N06C for ESTHER and N06AA, N06AB, N06AF, N06AG, N06AX for KORA). In order to assess the possible relationship of the epigenetic biomarker with a previous diagnosis of depression, sensitivity analyses were conducted in the ESTHER cohort with lifetime history of depression as response variable, which included both individuals who had suffered from a depressive episode in the past and individuals with prevalence of depression. Finally, sensitivity analyses were conducted with inclusion of different cell type sets in the regression models.

The EWAS and the KORA analyses were conducted with R (version 3.5.3); all other analyses were performed with SAS^®^ statistical software version 9.4 (SAS^®^ Institute Inc., Cary, NC, USA).

## Results

In the ESTHER cohort there were 2 254 participants with baseline DNA methylation and information on mental HRQOL. Out of this group *n* = 1 814 had information on the GDS-15 collected at the 5-year follow-up. After exclusion of participants with invalidated GDS (*n* = 95) and lifetime history of a physician diagnosis of depression (*n* = 246, thereof 19 with invalidated GDS), 1492 ESTHER participants remained for regression analyses. In the KORA cohort (KORA F4) there were 1 722 participants with both baseline DNA methylation and follow-up information (KORA FF4), but *n* = 821 did not have information on either prevalent or incident depressive symptoms as measured by the DEEX scale and were excluded from the study. Also, participants with possible baseline depression as measured by the PHQ-9 (*n* = 11) were excluded. Hence, 890 observations remained for replication analyses with the DEEX scale. As regards the KORA cohort no evidence of population stratification was found in multiple published analyses using KORA data^28^.

Mean baseline age was 63 years with a SD of 6.4 years in the ESTHER cohort and 56 years (SD 6.2) in the KORA cohort (Table 1). The mean value of the total score of the GDS-15 in the ESTHER cohort was 2.5 (SD 2.9; range 0–15) and the great majority of participants (*n* = 1292; 86.6%) had a total score ≤5. A small minority (*n* = 44; 3.0%) had a total score ≥11 and *n* = 156 (10.5%) had a total score higher than 5 and lower than 11. The mean value of MCS-12 scores was 47.9 (SD 9.2; range 15.3–66.9). In the KORA study, the mean DEEX score was 7.72 (SD 4.77; range 0–24). A large proportion of the sample was considered to have no signs of depressive symptoms as by the dichotomized DEEX scale (*n* = 592; 66.5%).

**Table 1:** Baseline characteristics of the ESTHER and KORA study population.

	ESTHER study *N* (%)	KORA study *N* (%)
***N*** **Total**	1492*	890*
**Age**		
(mean, SD)	62.5 (6.4)	55.7 (6.2)
**Sex**		
Women	748 (50.1)	456 (51.2)
Men	744 (49.9)	434 (48.8)
**Educational level**		
High^a^	150 (10.1)	214 (24.0)
Middle^b^	227 (15.2)	230 (25.8)
Low^c^	1115 (74.7)	445 (50.0)
**Cardiovascular disease**^**d**^		
No	1320 (90.6)	860 (96.6)
Yes	137 (9.4)	30 (3.4)
**Smoking**		
Never	686 (46.9)	341 (38.3)
Former	519 (35.4)	400 (44.9)
Current	259 (17.7)	149 (16.7)
**Physical activity**		
Yes^e^	516 (34.6)	333 (37.4)
No^f^	976 (65.4)	557 (62.6)
**Body mass index**		
(mean, SD)	27.7 (4.3)	27.4 (4.7)

*SD* standard deviation.

*Number of participants with both epigenetic measurements at baseline and follow-up information on depressive symptoms. Participants with lifetime history of depression (ESTHER) and with baseline depression (KORA) were excluded.

^a^General qualification for university entrance or higher qualification.

^b^Intermediate secondary school-leaving certificate.

^c^Certificate of completion of compulsory basic secondary schooling or lower.

^d^Combined endpoint of myocardial infarction or stroke. ESTHER: lifetime history; KORA: baseline prevalence.

^e^ESTHER: ≥2 h of physical activity/week; KORA: ≥1 h of physical activity/week.

^f^ESTHER: Inactive/<2h of physical activity/week; KORA: <1 h of physical activity/week.

In the ESTHER cohort epigenome-wide meta-analysis of MCS-12 based on subset I (*n* = 1000) and subset II (*n* = 864) revealed 5 CpGs reaching the epigenome-wide significance of *p*-value <1.0E−5 (cg13033086 *p*-value 9.77E−06; cg20256881 *p*-value 1.95E−06; cg24312390 *p*-value 8.21E−06; cg26693725 *p*-value 9.96E−06; cg27115863 *p*-value 8.55E−06). In subset III used to validate the 5 CpGs only cg27115863 remained significant with a *p*-value <0.05 (Table 2). The mean methylation value of cg27115863 was 0.40 (SD 0.04; range 0.09–0.53) both in the whole ESTHER sample (*n* = 1 814) and after exclusion of participants with lifetime history of depression (*n* = 1 492), with only a marginal change in the SD in the reduced sample (SD 0.05). In the KORA cohort the mean methylation value of cg27115863 was 0.39 (SD 0.05; range 0.13–0.52). As regards the blood cell types both in the ESTHER and in the KORA cohort the strongest correlations were observed between granulocyte and other four types of cells (NK, B cell, CD4T, and CD8T cell).

**Table 2:** Results of the epigenome-wide association study for subjective mental health—ESTHER cohort study (2000–2002).

CpG ID 27115863	Estimate	Standard error	*p*-Value
Epigenome-wide meta-analysis (subset I and subset II)	1.48E−06	3.30E−07	8.55E−06*
Replication set (subset III)	1.92E−06	7.01E−07	6.34–03*

Subjective mental health was measured with the Mental Component Summary of the SF-12.

*Raw *p*-value.

**Fig. 1:**
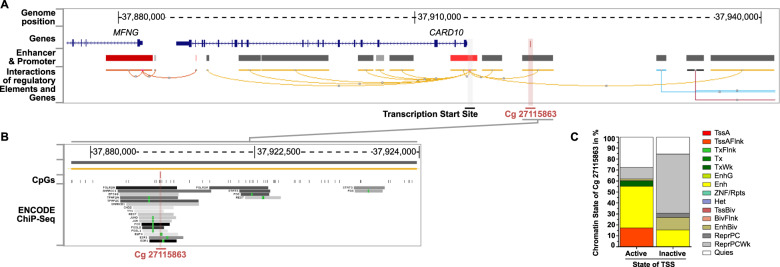
Cg27115863 is located in a putative regulatory region of the *CARD10* gene. Genome browser shot highlighting the genomic context of the Cg27115863 on Chromosome 22 (hg19/chr22:37921640–37921641). **a** The CpG is located approx. 6 kb proximal to the Transcription start site (TSS) of the *CARD10* gene. GeneHancer tracks (Enhancer (gray) & Promoter (red), Interactions between regulatory Elements and Genes) indicate that Cg27115863 is located within a putative enhancer region of the *CARD10* gene. **b** Zoom onto the GeneHancer element in which Cg27115863 is located. The CpG is located in a region of Transcription Factor binding sites indicated by the ENCODE ChiP-Seq track (161 Factors) with Factorbook Motifs (green)^42–44^. **c** Quantification of chromatin states of the Cg27115863 when active (State 1 – TssA) or inactive chromatin (State 13 – Repressed Polycomb, State 14 – Weak Repressed Polycomb, State 15 – Quiescent/low) marks where found at the TSS of CARD10 (hg19/chr22:37915549). The chromatin states (15 Primary Core Marks) of the Cg27115863 & TSS of *CARD10* in 127 tissues/cells where obtained from the Roadmap Epigenomics project (http://www.roadmapepigenomics.org/). Active TSS states can appear at enhancer regions due the close proximity of the TSS & enhancers in the 3D structure of the genome.

Cg27115863 is located on chromosome 22 (hg19/chr22:37921640–37921641) in an intergenic position approximal 6 kb far away from its closest gene *CARD10* (Fig. 1), which activates the nuclear factor kappa B (NF-kB)^29^. The GeneHancer tracks, which use multiple sources of data to link human regulatory elements (enhancers & promoters) to their inferred target genes^30^, indicate that cg27115863 is located within a putative enhancer region of the *CARD10* gene (Fig. 1a). The quantification of chromatin states at the cg27115863 position in 127 tissues/cells from the Roadmap Epigenomics project (http://www.roadmapepigenomics.org/) also provides further evidence indicating that the cg27115863 is located within a putative enhancer of the *CARD10* gene (Fig. 1c). Specifically, in tissues/cells with active TSS states at the TSS of the *CARD10* gene (hg19/chr22:37915549) a higher degree of enhancer states can be found at the position of the cg27115863 when compared to tissues/cells which display inactive states. This suggests that if the *CARD10* promoter is active the likelihood increases that the position of the cg27115863 is also marked as an enhancer.

Results of regression analyses in the ESTHER study indicated that hypomethylation of cg27115863 was associated with an increased risk of depressive symptoms over the observational period of 5 years (Table 3). An increase in methylation values was associated with a reduction in the odds of developing depressive symptoms at 5-year follow-up by 24% (OR_sd_ = 0.76; 0.63–0.91) in the ESTHER cohort. Participants with methylation beta values within the first quartile were nearly twice as likely to show depressive symptoms as compared to participants with methylation beta values within the fourth quartile (OR_Q1vsQ4_ = 1.95; CI 1.20–3.19 in fully adjusted model 2).

**Table 3:** Longitudinal association of subjective mental health and its epigenetic signature with depressive symptoms (ESTHER and KORA study).

	Logistic regressions: odds ratios (OR) with 95% confidence interval (CI) for incidence of depressive symptoms*	
	Model 1^a^ OR (CI)	Model 2^b^ OR (CI)
ESTHER cohort (*n* = 1492)		
**MCS-12 (per 10 units)**	0.46 (0.39–0.53)	0.46 (0.40–0.54)
**Cg27115863 (per SD)**	0.72 (0.61–0.86)	0.76 (0.63–0.91)
**Cg27115863 (quartiles)**		
Q1 vs. Q4	2.20 (1.37–3.54)	1.95 (1.20–3.19)
Q2 vs. Q4	1.30 (0.80–2.12)	1.29 (0.78–2.14)
Q3 vs. Q4	1.53 (0.96–2.44)	1.48 (0.91–2.39)
KORA cohort (*n* = 890)		
**Cg27115863 (per SD)**	0.84 (0.69–1.02)	0.84 (0.69–1.03)
**Cg27115863 (quartiles)**		
Q1 vs. Q4	1.66 (0.95–2.89)	1.62 (0.92–2.87)
Q2 vs. Q4	1.58 (0.95–2.64)	1.53 (0.91–2.57)
Q3 vs. Q4	1.30 (0.80–2.14)	1.28 (0.78–2.12)
Meta-analysis of ESTHER and KORA		
**Cg27115863 (per SD)**	0.77 (0.66, 0.89)	0.80 (0.70, 0.91)
**Cg27115863 (quartiles)**		
Q1 vs. Q4	1.96 (1.36, 2.81)	1.80 (1.25, 2.61)
Q2 vs. Q4	1.43 (1.00, 2.03)	1.40 (0.98, 2.01)
Q3 vs. Q4	1.42 (1.01, 1.98)	1.38 (0.97, 1.95)

MCS-12, Mental Component Summary of the Short-Form 12 Health Survey; SD standard deviation.

*Depressive symptoms were measured with the Geriatric Depression Scale (GDS) in the ESTHER and with the Depression and Exhaustion Scale (DEEX) in the Kora cohort. A GDS score > 5 and a DEEX score ≥9 in men and ≥11 in women were taken as cut-off for depressive symptoms.

^a^Model 1 adjusted for age, sex, educational level. Model 1 including cg27115863 also adjusted for batch effects and leukocyte distribution in both cohorts and for the DEEX as measured at baseline in the KORA cohort.

^b^Model 2 additionally adjusted in both cohorts for smoking, physical activity, body mass index, and lifetime history of cardiovascular disease (KORA: baseline prevalence).

In the KORA cohort similar association patterns as those found in the ESTHER cohort were observed. Results indicated an inverse association between methylation of cg27115863 and incidence of depressive symptoms (OR_sd_ = 0.84; CI 0.69–1.03; OR_Q1vsQ4_ = 1.62; CI 0.92–2.87), but confidence intervals included 1.0. However, results of the meta-analysis conducted with the results obtained with both cohorts further confirmed and reinforced the findings (OR_sd_ = 0.80; CI 0.70–0.91; OR_Q1vsQ4_ = 1.80; CI 1.25–2.61).

In both cohorts the results remained stable after additional exclusion of participants using antidepressants at baseline (ESTHER: n = 57 excluded; OR_Q1vsQ4_ = 1.91; CI 1.16–3.16; KORA: n = 28 excluded; OR_Q1vsQ4_ = 1.74; CI 0.98, 3.11 in fully adjusted models). In the ESTHER cohort further sensitivity analyses conducted with lifetime history of a physician diagnosis of depression as response variable showed that the epigenetic marker was not associated with lifetime history of depression (results not shown). Finally, in the ESTHER cohort after excluding granulocyte from the set of cell types the regression coefficients of the candidate CpGs remained stable and only changed in the third decimal place. In the KORA cohort the inclusion of all six estimated cell types did not change the results.

As regards mental HRQOL for 10-unit increase in MCS-12 scores the odds for depressive symptoms at 5-year follow-up were reduced by 54% (OR 0.46; CI 0.40–0.54) in the fully adjusted regression model 2 and the association remained stable even after addition of the epigenetic marker (OR 0.45; CI 0.39–0.54) or the additional exclusion of participants using antidepressants (OR 0.47; CI 0.40–0.55).

## Discussion

In this longitudinal study we found that poor mental HRQOL is associated with future depressive symptoms among people with no lifetime history of depression and that possible epigenetic and inflammatory mechanisms might underpin such an association. These findings show that self-perceived mental HRQOL and its epigenetic signature have the potential to predict depressive symptoms in later life. Specifically, poor mental HRQOL might leave an epigenetic signature, which might contribute to depressive symptoms through the activation of inflammatory pathways. The replication of the analyses in an independent cohort and the results of the meta-analysis strengthen the results indicating that hypomethylation at cg27115863 might be a risk factor for future depressive symptoms. The lack of association between the epigenetic marker and lifetime history of a physician diagnosis of depression reinforces the predictive value of the marker for depressive symptoms in later life.

The location of cg27115863 in a putative enhancer region of the gene *CARD10*, which activates NF-kB, a transcription factor in inflammatory signaling that is involved in the pathophysiology of depression^31^, supports the role of inflammation in the development of depressive symptoms in later life. Specifically, it has been found that stress activates NF-kB signaling and in turn NF-kB mediates depressive-like behavior caused by stress^32^. MCS-12 scores represent a subjective measure of general mental health that is not specific to any disease or treatment group and that reflects, inter alia, psychological distress and psychological wellbeing^9,33^. Hence, it might be speculated that difficulties in coping with daily stressors activate inflammation pathways through gene *CARD10* and this gene × environment interaction might leave an epigenetic signature through cg27115863, which in turn might predispose to depression. This interpretation is also supported by evidence showing that the aged brain retains both plasticity and vulnerability to psychosocial stress^34^.

Methylation differences at cg27115863 were also reported in an epigenome-wide association study conducted among cases with schizophrenia and controls^35^. That cg27115863 has been found differently methylated in an EWAS for schizophrenia is of particular interest since there is evidence for a shared genetic and biological basis for major depression and schizophrenia^36^. However, given the small effect estimates for cg27115863, the results based on methylation analyses should be interpreted with caution and further studies shall assess whether the methylation status is changing substantially.

Another relevant result of the study is the predictive value of mental HRQOL for depressive symptoms. Previous cross-sectional studies showed that MCS-12 scores could be useful in identifying prevalence of depression^37–39^. Our study adds to this by indicating that mental HRQOL comprises distinctive features that could predict the development of depressive symptoms even among older people free of lifetime history of depression. Hence, the MCS-12 might be used as a screening instrument for older people and cg27115863 would offer a biological “counterpart” to the instrument. The focus on depressive symptoms in later life strengthens the results because it is likely to limit the potential heterogeneity involved in the clinical presentation and pathophysiology of depression. However, it would be necessary to assess whether cg27115863 is specific for depressive symptoms or whether it is associated with other pathologies that are known to be associated with depression and inflammation markers, such as cardiovascular disease. In this study cg27115863 had only a predictive value for depressive symptoms, but the specificity of cg27115863 for depressive mood needs further validation. Moreover, several studies found a relationship between personality characteristics and health-related quality of life with neuroticism being related to poorer self-perceived quality of life, including mental HRQOL^40^. Hence, the explanatory role of HRQOL independently of personality characteristics should also be further explored.

The much smaller sample size of the KORA cohort (*n* = 890) as compared to the ESTHER cohort (*n* = 1 492) most likely contributes to explaining the lack of statistical significance in the KORA results. Additionally, in the KORA and ESTHER study the predictive value of cg27115863 was assessed in two different screening instruments for depression (the DEEX scale and the GDS-15, respectively), with the DEEX, differently from the GDS-15, focusing both on exhausted and depressed mood and not being specifically designed for detecting depressive symptoms among older people like the GDS-15. However, this might be an indication of the robustness of our findings since the results indicating hypomethylation as a risk factor were similar despite the use of different instruments and different cohorts. Also, the results of the meta-analysis further reinforce the findings.

A limitation of this study is the missing causal role of cg27115863 in the gene expression regulation of *CARD10*, its closest gene, which cannot be tested in this epidemiologic study and will have to be addressed by future functional studies. Another limitation of the study is that lifetime history of a medical diagnosis of depression, including prevalence of depression at baseline, was self-reported in the ESTHER study, hence misclassification due to recall bias or social desirability bias might have occurred. In the ESTHER cohort epigenetic measurements were performed in three different subsets in the context of different sampling strategies. However, this should not have a major effect on the results because the variation in the demographic composition was accounted for in the regression models through adjustment for the relevant covariates. Given that the vast majority of the study population were Caucasians, we did not specifically adjust for genetic ancestry. Nevertheless, we adjusted for blood cell composition throughout all analyses, and adjustment for blood cell composition has been suggested to yield essentially the same results as controlling for genetic ancestry in DNA methylation studies^41^.

The replication of the results through an independent cohort and the performance of the meta-analysis were important strengths of this study. Other strengths were the large sample size and the longitudinal design.

In conclusion, this work shows that subjective mental health and its epigenetic “counterpart” might be a useful instrument to identify older people at risk of developing depressive symptoms. Also, it suggests that inflammatory pathways might have an epigenetic underpinning contributing to the pathogenesis of depressive symptoms in later life. However, the predictive value of mental HRQOL and the exact role of cg27115863 and NF-kB for depressive mood needs further validation both in epidemiologic cohorts and *in vitro*.

## Supplementary information

Supplemental Figure 1
