# Supplementary figures and images for: Subjective mental health, incidence of depressive symptoms in later life, and the role of epigenetics: results from two longitudinal cohort studies
Source: Transl Psychiatry. 2020 Sep 21;10:323. doi: 10.1038/s41398-020-00997-x (PMC7506005; doi:10.1038/s41398-020-00997-x)

**Supplemental Figure 1 Study design**

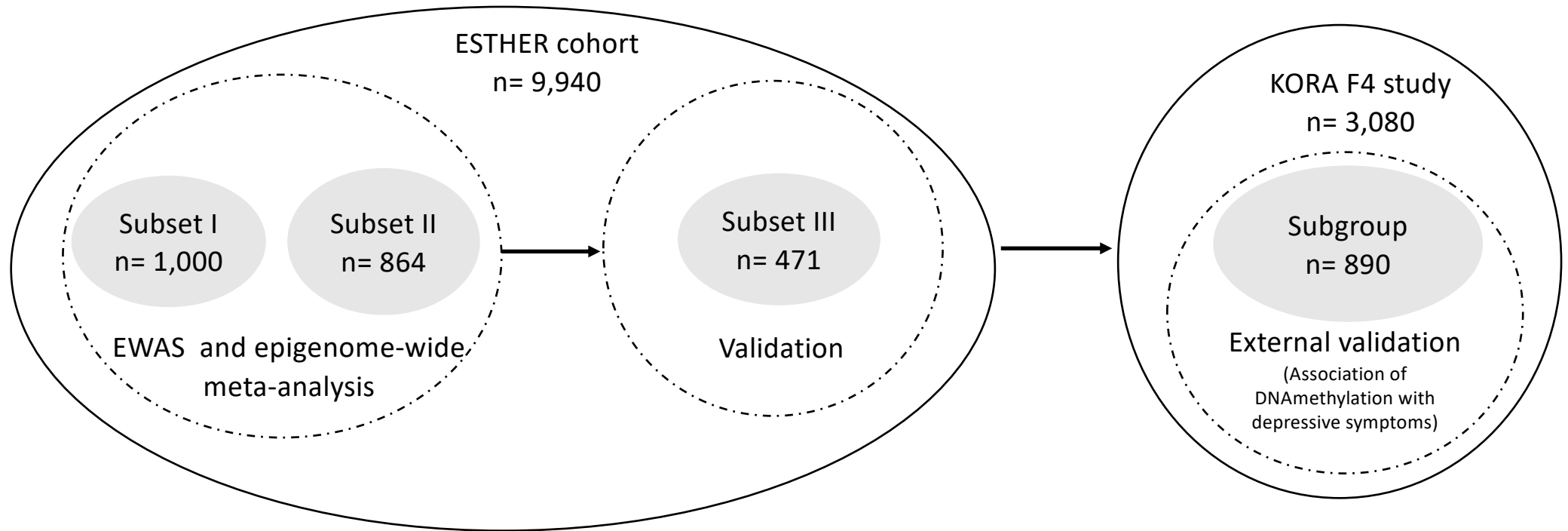

Supplement: Supplementary file 1 — Supplemental Figure 1 [file 41398_2020_997_MOESM1_ESM.pdf]
